# Dual Functions of V/SiO_*x*_/AlO_*y*_/p^++^Si Device as Selector and Memory

**DOI:** 10.1186/s11671-018-2660-9

**Published:** 2018-08-23

**Authors:** Sungjun Kim, Chih-Yang Lin, Min-Hwi Kim, Tae-Hyeon Kim, Hyungjin Kim, Ying-Chen Chen, Yao-Feng Chang, Byung-Gook Park

**Affiliations:** 1 School of Electronics Engineering, Chungbuk National University, Cheongju, 28644 Republic of Korea; 2 Department of Physics, National Sun Yat-sen University, Kaohsiung, 804 Taiwan; 3 Department of Electrical and Computer Engineering, Inter-University Semiconductor Research Center (ISRC), Seoul National University, Seoul, 08826 South Korea; 4 Department of Electrical and Computer Engineering, Microelectronics Research Center, University of Texas at Austin, Austin, TX 78758 USA; 5 Intel Corporation, Hillsboro, USA

**Keywords:** Resistive switching, Selector, Memory, Nonlinearity, Silicon oxide, Vanadium

## Abstract

This letter presents dual functions including selector and memory switching in a V/SiO_*x*_/AlO_*y*_/p^++^Si resistive memory device by simply controlling compliance current limit (CCL). Unidirectional threshold switching is observed after a positive forming with low CCL of 1 μA. The shifts to the V-electrode side of the oxygen form the VO_*x*_ layer, where the threshold switching can be explained by the metal-insulation-transition phenomenon. For higher CCL (30 μA) applied to the device, a bipolar memory switching is obtained, which is attributed to formation and rupture of the conducting filament in SiO_*y*_ layer. 1.5-nm-thick AlO_*y*_ layer with high thermal conductivity plays an important role in lowering the off-current for memory and threshold switching. Through the temperature dependence, high-energy barrier (0.463 eV) in the LRS is confirmed, which can cause nonlinearity in a low-resistance state. The smaller the CCL, the higher the nonlinearity, which provides a larger array size in the cross-point array. The coexistence of memory and threshold switching in accordance with the CCL provides the flexibility to control the device for its intended use.

**Electronic supplementary material:**

The online version of this article (10.1186/s11671-018-2660-9) contains supplementary material, which is available to authorized users.

## Background

Resistive random-access memory (RRAM) is one of the promising candidates for the next-generation non-volatile memory technology due to its fast switching speed [1, 2], low-power consumption [3–8], multilevel capability [9–15], high scalability [16–20], and 3D stacking ability [21–25]. These properties are especially suitable for storage class memory (SCM) which can fill the performance gap between dynamic random-access memory (DRAM) as a main memory and solid-state-drive (SSD) as a storage memory. Even though RRAM device has made much progress in the past years [1–25], there remains, however, a major disadvantage: sneak current through neighboring cells occurs in a high-density cross-point array [26]. The memory device with the selector component should provide nonlinear current–voltage (I–V) characteristics to overcome this problem [26–35]. Until now, various devices with nonlinear concepts such as complementary resistive switching (CRS) [26], tunnel barrier [27–33], Ag-based threshold switching [34], diode-type selector [35, 36], ovonic threshold switching (OTS) [37, 38], and metal-insulator-transition (MIT) [39–43] have been reported. VO_*x*_ as one of the typical MIT materials could be widely used in potential applications as optical and electrical switching component [40–42]. SiO_2_ is widely used as a passivation layer in the semiconductor industry. Moreover, Si-rich SiO_*x*_ (*x* < 2) can be used as a resistance change layer in RRAM [44–55]. SiO_*x*_ can be preferred over many other materials in terms of compatibility with CMOS processes and low cost. SiO_*x*_-based RRAM devices have been reported to act as a mediator of the role of conducting bridges simply by using electrodes such as Cu and Ag with high diffusivity [44–47]. In another case, memory switching is induced by the valence change effect inside the SiO_*x*_ layer, which can be explained by generation of oxygen vacancies or proton exchange model [48–55]. In the unipolar switching where a set operation precedes a reset, it is sensitive to the ambient atmosphere. The switching performance in the air is significantly degraded [48–53]. On the other hand, filamentary switching without backward-scan effects shows typical unipolar and bipolar switching in various SiO_*x*_-based RRAM devices [52–54].

Here, we present the coexistence of threshold switching and memory switching in V/SiO_*x*_/AlO_*y*_/p^++^Si device depending on compliance current limit (CCL). The device with silicon bottom electrode (BE) has several advantages compared to the conventional metal electrode. The RRAM device with memory or threshold switching is directly connected to the source or drain side in a transistor, which is a potential application for embedded memory and steep slope device. The overshoot current could be reduced due to the series resistance of Si BE. Moreover, nano-tip of silicon BE through wet etching and the adjustment of the doping concentration in silicon surface can improve switching performance. The AlO_*y*_ layer, which is a large band gap with an insulated property, helps to lower the operating current during threshold and memory switching. The SiO_*x*_ layer acts as memory switching layer at a high CCL, while it serves to supply oxygen to V TE at low CCL, which provides threshold switching.

## Methods

V/SiO_*x*_/AlO_*y*_/p^++^Si device was fabricated as follows: Firstly, BF_2_ ions were implanted with an acceleration energy of 40 keV and a dose of 5 × 10^15^ cm^−2^ into a Si substrate to heavily doped Si BE. The lattice damage was cured by the annealing process at 1050 °C for 10 min. Heavily doped Si BE had sheet resistance of 30.4 Ω/□. Next, a 1.5-nm-thick AlO_*y*_ layer was deposited by an atomic layer deposition (ALD) system using H_2_O and Al (CH_3_)_3_ and a 5.5-nm-thick SiO_*x*_ layer underwent plasma-enhanced chemical vapor deposition (PECVD) by reacting 5% SiH_4_/N_2_ (160 sccm), N_2_O (1300 sccm), and N_2_ (240 sccm) at 300 °C. Subsequently, a 50-nm-thick vanadium (V) top electrode (TE) with a diameter of 100 μm was deposited by DC sputtering a V target with Ar gas (30 sccm). Finally, a 50-nm-thick Al as a protective layer was deposited by DC sputtering to prevent further oxidation of V TE. All electrical properties were characterized via the DC voltage sweep and pulse modes using a Keithley 4200-SCS semiconductor parameter analyzer (SPA) and a 4225-PMU ultra-fast current–voltage (I–V) module at room temperature, respectively. For device operation, the TiN BE was grounded and the Ni TE bias was controlled.

## Results and Discussion

**Fig. 1:**
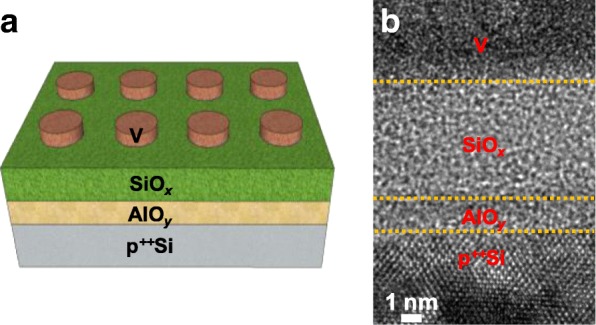
Device configuration of V/SiO_*x*_/AlO_*y*_/p^++^Si. **a** Schematic drawing and **b** TEM image

**Fig. 2:**
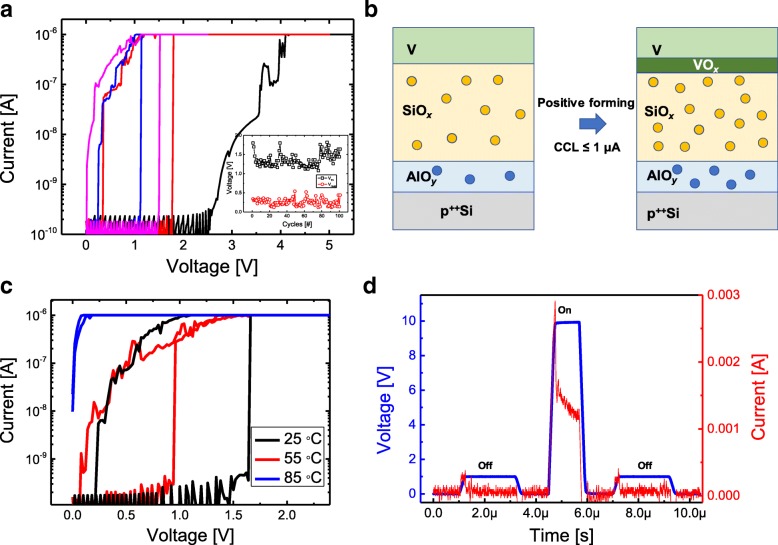
Unidirectional threshold switching of V/SiO_x_/AlO_y_/p^++^Si when a positive forming with CCL of 1 μA is applied. **a** Typical I–V curves. **b** Schematic drawing of forming process. **c** I–V characteristics by temperature dependence. **d** Transient characteristics

Figure 1a shows the schematic structure of V/SiO_*x*_/AlO_*y*_/p^++^Si device. Three amorphous V, SiO_*x*_, and AlO_*y*_ layers and single-crystalline Si layer are observed by a transmission electron microscopy (TEM) cross-sectional image as shown in Fig. 1b. The thicknesses of the SiO_*x*_ and AlO_*y*_ layers are 5.5 and 1.5 nm, respectively. To confirm the composition ratio of two dielectric films, XPS analysis was conducted (Additional file 1). The *x* value of SiO_*x*_ and the *y* value of AlO_*y*_ are 0.88 and 1.33, respectively. Our SiO_*x*_ film using PECVD compared to SiO_2_ deposited using dry oxidation is deposited at a much lower temperature and has much more defects, making them suitable for resistive switching at relatively lower voltages. Figure 2a shows typical threshold switching of V/SiO_*x*_/AlO_*y*_/p^++^Si device. The initial switching with a positive forming process requires higher voltage than subsequent threshold switching since the dielectric layers have initially smaller defects. A CCL of 1 μA is applied to the device to avoid the formation of excessive conducting filaments in the SiO_*x*_ layer. The leakage current is very low (100 pA at 1 V) compared with previously reported threshold switching of VO_*x*_. This advantage is attributed to the Al_2_O_3_ with higher permittivity and thermal conductivity compared to the SiO_2_. Off-state has the insulating property because the filaments are easily ruptured and then there are no remaining filaments. A possible mechanism for threshold switching is the oxidation of the V TE from the oxygen supplied from the SiO_*x*_ layer during the positive forming process as shown in Fig. 2b. The electrical property of VO_*x*_ between V TE and SiO_*x*_ layer may change from insulating state to metallic state, causing a sudden change in resistance. A low CCL of 1 μA is not sufficient to cause efficient conducting filaments inside the SiO_*x*_ film. Therefore, SiO_*x*_ with insulating properties can be another cause to reduce the off-current. For a negative forming process of V/SiO_*x*_/AlO_*y*_/p^++^Si device, a threshold switching is not observed (see Additional file 1). When the negative bias is applied to the V TE, the movement of the oxide moves toward the Si BE, so that the V TE can no longer participate in the threshold switching as VO_*x*_. Inset of Fig. 2a exhibits the threshold voltage (V_th_) and hold voltage (V_hold_) during the 100 cycles. The V_th_ where the current sharply increases with nearly infinite slope is between 1.08 and 1.82 V, and V_hold_ at which point the current return to a high-resistance state is between 0.12 and 0.54 V. Figure 2c shows the I–V characteristics in the on-current at different temperatures. At 25 °C and 55 °C, they show almost similar threshold switching, but I–V curve at a higher temperature of 85 °C loses the threshold switching property. It is well known that VO_*x*_ loses its MIT at high temperatures. Thus, this result is another proof that VO_*x*_ is the main cause of the threshold switching. Figure 2d shows the transient characteristics for threshold switching. The pulse with the amplitude of 1 V monitored the read current before and after writing pulse with width of 1 μs. The high current was monitored while the pulse with high amplitude is applied to the device, and then, the V/SiO_*x*_/AlO_*y*_/p^++^Si device turned off the current immediately after the writing pulse was removed. The selector properties analyzed above can be used when combined with operation of memory elements below 1 μA [55, 56].

**Fig. 3:**
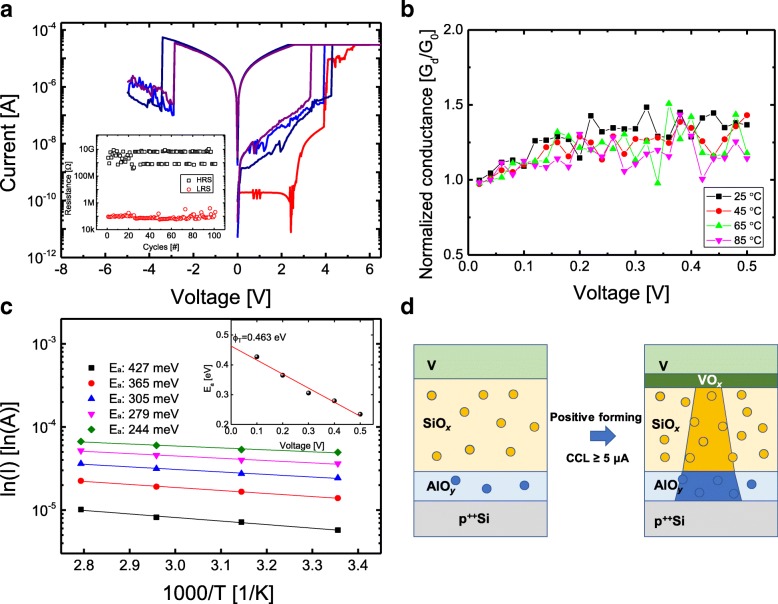
Memory switching of V/SiO_*x*_/AlO_*y*_/p^++^Si when a positive forming with CCL of 30 μA is applied. **a** Typical I–V curves. **b** Normalized conductance. **c** In (I) versus 1000/T. **d** Schematic drawing of forming process

Figure 3a shows the bipolar resistive switching of V/SiO_*x*_/AlO_*y*_/p^++^Si device after a positive forming with CCL of 100 μA. Then, the reset process with a rapid increase in resistance is performed by sweeping the negative voltage, and the device is switched to a high-resistance state (HRS). The set process with a rapid decrease in resistance then occurs at a positive bias voltage, causing the device to turn back to a low-resistance state (LRS). In order to understand the properties of the conducting filament, we observe the normalized conductance and the temperature dependence. The conduction in the LRS is an important guideline to indirectly inform the properties of the conducting filament. Figure 3b shows the normalized conductance (G_N_) which is defined as the dynamic conductance (G_d_) divide by static conductance (G_0_) for I–V curves of V/SiO_*x*_/AlO_*y*_/p^++^Si device in the LRS with different temperatures. Regardless of the temperature, the G_N_ value converges to 1 when the voltage is zero. This allows us to rule out the well-known conduction mechanism such as Schottky emission, Fowler-Nordheim tunneling, and Child’s law (I~V^2^) in space-charge-limited current (SCLC). Metallic ohmic conduction can also be excluded considering temperature dependence as shown in Fig. 3c. The decrease in resistance with increasing the temperature suggests that the conducting filament has a semiconducting property. Thus, we can exclude the penetration of V into the SiO_*x*_ layer for the main conducting filament of V/SiO_*x*_/AlO_*y*_/p^++^Si device in LRS. Therefore, the bipolar memory operation of the V/SiO_*x*_/AlO_*y*_/p^++^Si device is dominated by intrinsic switching of SiO_*x*_. It is also confirmed that the positive and negative currents are not that much different suggesting that rather than an interface-type such as Schottky emission, it is dominated by bulk conduction. Taking into account the abovementioned normalized conductance, there are two possible bulk dominant conduction mechanisms. The first one is hopping conduction following the formula:

$$ J={qnav}_o{e}^{-q{\o}_T/ kT}{e}^{qaV/2 dkT} $$

where *q*, *n*, *a*, *ø*_*T*_, *v*_*o*_, and *d* are the electric charge, concentration of space charge, mean of hopping distance, electron barrier height for hopping, intrinsic vibration frequency, and the thickness of dielectric film, respectively. The *ø*_*T*_ calculated from the slope of a linear plot of ln (I) versus 1000/T is 0.463 eV as shown in Fig. 3c. A value calculated from the relationship between E_a_ and V is 5.17 nm, indicating the conducting filament formed in the SiO_*x*_ is not strong and is close to the HRS state. The other conduction mechanism, the Poole-Frenkel (P-F) emission, was covered in Additional file 1. Based on the above results, the conducting filament model in the memory operation of V/SiO_*x*_/AlO_*y*_/p^++^Si device is depicted in Fig. 3d. In the positive forming process, the oxidation process proceeded on the V TE side, but due to the high CCL, a conductive filament can be formed inside the SiO_*x*_ and AlO_*y*_ due to the movement of the oxygen vacancies. During the reset process, the electric field opposite to the forming and set induces oxygen and recombination with the oxygen vacancy, resulting in the rupture of the conductive filament. It is noted that the selector and memory operations are observed in the same cell. Memory operation is possible after the threshold operation has occurred and then the switch is completely turned off. However, the reverse direction is not possible because the reset switching of the memory operation is not completely turned off.

**Fig. 4:**
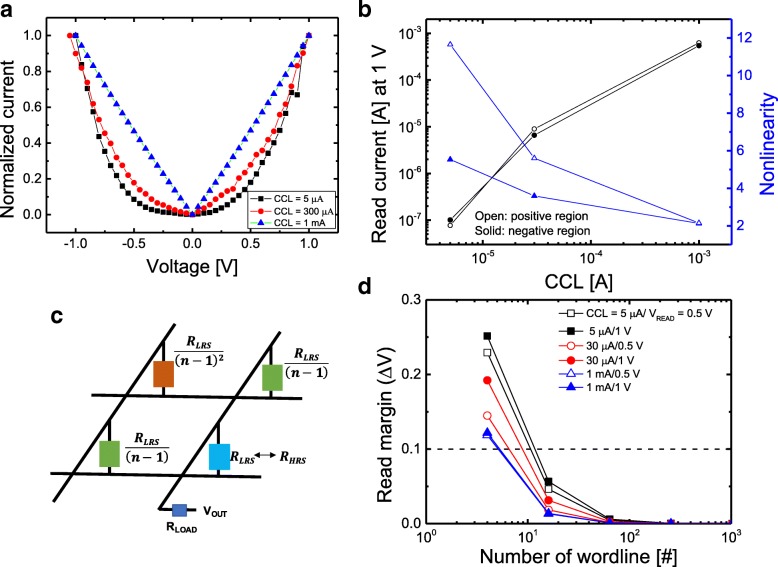
Nonlinear characteristics of V/SiO_*x*_/AlO_*y*_/p^++^Si for memory switching. **a** I–V curves with different CCLs. **b** Read current and nonlinearity as functions of CCL. **c** Equivalent circuits of cross-point array. **d** Read margin as a function of word line number for different CCLs and read voltage

Figure 4a shows normalized I–V curves in the LRS of V/SiO_*x*_/AlO_*y*_/p^++^Si device at low-voltage regime (0~1 V) for different CCL conditions (5 μA, 30 μA, and 1 mA). Here, the normalized I–V curve is defined as the current at each voltage divided by the current at 1 V. Since the levels of the LRS current depending on the CCL are varied, we set the current value at 1 V to easily compare the nonlinearity. It can be observed that as the CCL decreases, the current is suppressed at lower voltage regime. In order to derive a more quantitative relation, nonlinearity is defined as the ratio of the current at V_READ_ to that at the half of V_READ_. Figure 4b shows the read current at 1 V and nonlinearity as a function of CCL for V/SiO_*x*_/AlO_*y*_/p^++^Si device. The decrease in read current due to CCL reduction suggests that the conducting filament is becoming finer and then the nonlinearity increases. The intrinsic silicon oxide film exhibits high nonlinearity even in a single layer. The intrinsic nonlinear property is due to the bulk nature of the silicon oxide rather than the interface of the silicon. The smaller the CCL is, the less the degradation is generated in the SiO_*x*_, so the lowering of the trap energy level in the LRS compared to that in the HRS can be minimized. Therefore, the higher energy barrier can maximize nonlinearity in the LRS state when lower CCL is applied to the device. Similarly, the conduction described by the P-F emission in the TaO_*x*_/TiO_*y*_ stack ensures high nonlinearity [57]. Another possibility is that because the dielectric constant of the oxide is smaller, more passes are made to the oxide film due to the concentration of the field. This can lead to the lowering of the trap energy level of the oxide layer, which can be expected to serve as a tunnel barrier for Al_2_O_3_. To obtain to the read margin (∆V) in *n* × *n* cross-point array, we use the simplified equivalent circuit as shown in Fig. 4c. Considering the worst case, the adjacent cells are set to the LRS and the load resistance (R_L_) to the LRS resistance. The ∆V was calculated from difference between V_OUT_ at LRS and V_OUT_ at HRS. Figure 4d shows the ∆V as a function of number of word lines (*n*) for V/SiO_*x*_/AlO_*y*_/p^++^Si device. The smaller the CCL, the higher the ∆V because the nonlinearity increases. When 10% read margin is secured, the array can be expanded to about more than 10 × 10 for CCL of 5 μA and to 5 × 5 for CCL of 1 mA. The array size to withstand the sneak current is not sufficient, but it will help expand the array size when the device with selector function is connected in a V/SiO_*x*_/AlO_*y*_/p^++^Si device. Compared to 0.5-V read in all CCLs, it has higher nonlinearity with read at 1 V. Although low V_READ_ leads to low static power in the read operation, the value of nonlinearity becomes smaller, which is due to the fact that the electric field is less on the SiO_*x*_/AlO_*y*_ layer in smaller V_READ_.

## Conclusions

In this work, a V/SiO_*x*_/AlO_*y*_/p^++^Si device having both a selector and a memory function by simply controlling CCL is investigated. When a CCL of 1 μA or less is applied, unidirectional threshold switching is observed for selector application. Positive forming oxidizes the V electrode and the MIT phenomenon of VO_*x*_ can induce threshold switching. The AlO_*y*_ layer is able to achieve a high selectivity of 10^4^ by lowering the off-current. On the other hand, when a CCL of 5 μA or more is applied, memory switching is observed as effective conducting filaments are formed on the SiO_*x*_ layer. The lower the CCL, the greater the nonlinearity, which helps to increase the size of the cross-point array.

## Additional File

Additional file 1: Supporting information. (DOCX 81 kb)

## Abbreviations

ALD: Atomic layer deposition
BE: Bottom electrode
CCL: Compliance current limit
CRS: Complementary resistive switching
DRAM: Dynamic random-access memory
HRS: High-resistance state
I–V: Current–voltage
LRS: Low-resistance state
MIT: Metal-insulator-transition
OTS: Ovonic threshold switching
PECVD: Plasma-enhanced chemical vapor deposition
P-F: Poole-Frenkel
RRAM: Resistive random-access memory
SCLC: Space-charge-limited current
SCM: Storage class memory
SPA: Semiconductor parameter analyzer
SSD: Solid-state-drive
TE: Top electrode
TEM: Transmission electron microscopy
V: Vanadium
