# Supplementary material for: Dual Functions of V/SiOx/AlOy/p++Si Device as Selector and Memory
Source: Nanoscale Res Lett. 2018 Aug 23;13:252. doi: 10.1186/s11671-018-2660-9 (PMC6107449; doi:10.1186/s11671-018-2660-9)
Supplement: Supplementary file 1 — Supporting information. (DOCX 81 kb) [file 11671_2018_2660_MOESM1_ESM.docx]

**Dual functions of V/SiO*_x_*/AlO*_y_*/p^++^Si device as selector and memory**

Sungjun Kim^1^, Chih-Yang Lin^2^, Min-Hwi Kim^3^, Tae-Hyeon Kim^3^, Hyungjin Kim^3^,

Ying-Chen Chen^4^, Yao-Feng Chang^5^*, and Byung-Gook Park^3^*

*^1^School of Electronics Engineering, Chungbuk National University, Cheongju 28644, Republic of Korea*

*^2^Department of Physics, National Sun Yat-sen University, Kaohsiung 804, Taiwan*

*^3^Department of Electrical and Computer Engineering, Inter-University Semiconductor Research Center (ISRC), Seoul National University, Seoul 08826, South Korea*

*^4^Department of Electrical and Computer Engineering, Microelectronics Research Center, University of Texas at Austin, Austin, Texas 78758, USA*

*^5^Micron Technology, Inc., Boise, ID 83716, USA*

To analyze the SiO*_x_* and AlO*_y_* films deposited in our laboratory. X-ray photoelectron spectroscopy (XPS) analysis was performed by using a Thermo VG ESCA Sigma Probe spectrometer operated at 15 kV and 100 W with a monochromatric Al-Ka radiation source. The calibration of the binding-energy scale was set by fixing the C 1s at 284.5 eV.

Figure S1 XPS spectra of SiO*_x_* film: Si 2p (a) and O 1s (b) and AlO*_y_* film: Al 2p (c) and O 1s (d).

Figure S2 Negative forming of V/SiO*_x_*/AlO*_y_*/p^++^-Si device.
